# Cutaneous Post-transplant Lymphoproliferative Disorder

**DOI:** 10.7759/cureus.63951

**Published:** 2024-07-06

**Authors:** Alpa Kanji, Raida Ahmad, Laksha Bala, Eleanor Mallon

**Affiliations:** 1 Dermatology, Imperial College Healthcare NHS Trust, London, GBR; 2 Pathology, Imperial College Healthcare NHS Trust, London, GBR

**Keywords:** post-transplant lymphoproliferative disorders, immunosuppressed, rituximab, transplant, ulcer

## Abstract

Post-transplant lymphoproliferative disorders (PTLD) involve T- or B-cell proliferation in an immunosuppressed transplant recipient. It usually presents at extra-nodal sites and can affect several organs. Cutaneous manifestations of PTLD are relatively rare and can be very heterogeneous.

We report a case of a 36-year-old male cardiac transplant recipient on long-term immunosuppression (ciclosporin, azathioprine, and prednisolone) who presented with a three-month history of a painless ulcer on the right lower leg. A skin biopsy showed a dermal atypical lymphoid infiltrate positive for PAX5, CD20 and MUM1 on immunohistochemistry and EBV with in-situ hybridisation and a 70% Ki-67 cell proliferation index. A whole body fluorodeoxyglucose (FDG) positron emission tomography (PET) scan showed increased tracer uptake corresponding to the site of the cutaneous ulcer, the anterior cortex of the right lower tibia, an area adjacent to the right superficial femoral artery and the right inguinal node.

These findings were in keeping with monomorphic B-cell post-transplant lymphoproliferative disorder (PTLD) consistent with diffuse large B-cell lymphoma, non-germinal centre subtype. Cessation of azathioprine and treatment with an anti-CD20 antibody, rituximab, led to clinical resolution of the ulcer and a negative FDG-PET scan, with no disease recurrence to date.

We present a rare case of monomorphic PTLD with cutaneous involvement, presenting with a solitary, painless ulcer, which was successfully treated with a reduction in immunosuppression and additional rituximab monotherapy, given the aggressive subtype. PTLD can arise many years post-transplant and is a serious, potentially life-threatening complication. Therefore, early recognition and prompt treatment are of paramount importance.

## Introduction

Post-transplant lymphoproliferative disorders (PTLD) are lymphomas arising in immunosuppressed transplant patients, in both solid transplant and haematopoietic stem cell recipients, and can be a serious and sometimes fatal complication. They can be considered a spectrum of disorders involving lymphoproliferative processes of B- and T-cells. The majority of cases are associated with Epstein-Barr virus (EBV) infection and involve B-cell proliferation. As T-cell activity is suppressed in the immunosuppressed state, this is thought to alter immune surveillance, resulting in the proliferation of latently infected B-cells. Proliferation of a malignant B-cell clone results in a lymphoma [1,2]. However, the role of EBV is not fully understood, as EBV-negative cases have been reported [3]. PTLD can be heterogeneous in presentation where cutaneous manifestations are relatively rare. We present the case of a patient with PTLD with cutaneous involvement, which highlights the need for dermatologists to maintain a high index of suspicion of this entity in the context of an immunosuppressed transplant recipient.

## Case presentation

A 36-year-old man presented with a three-month history of a painless ulcer on the right lower leg. He had a complex medical background; he had received a cardiac transplant four years ago secondary to congenital heart disease and had since been on long-term immunosuppression (ciclosporin, azathioprine and prednisolone). He was receiving haemodialysis for end-stage renal failure.

**Figure 1:**
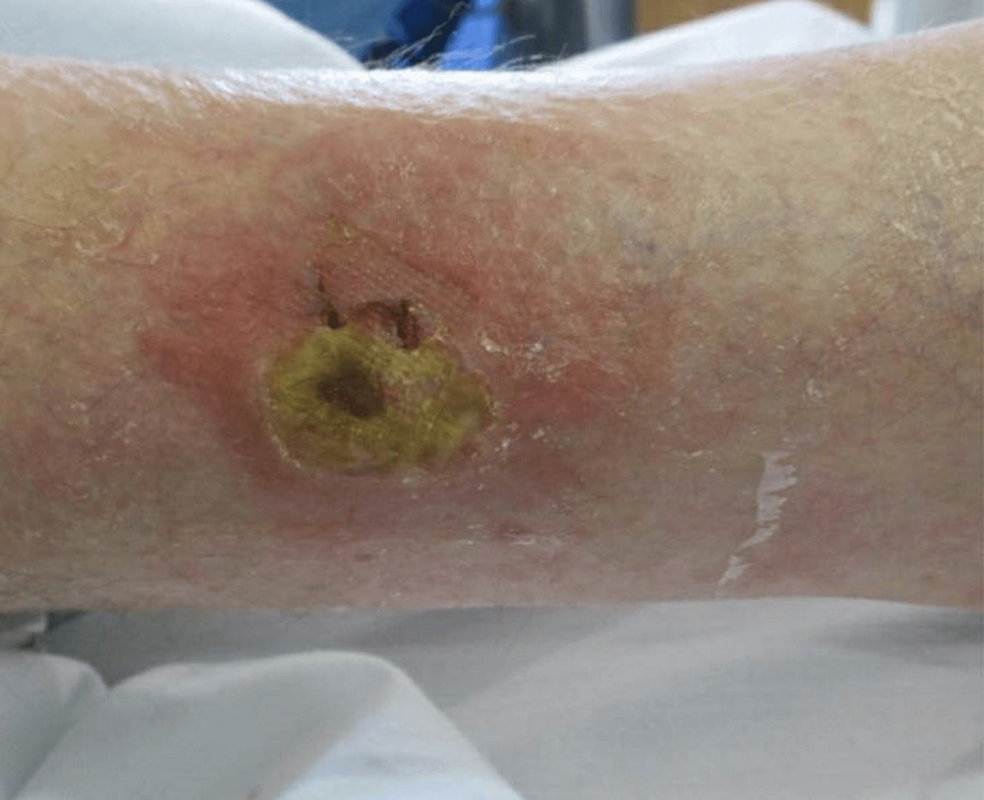
Patient photograph A 4 cm x 5 cm ulcer on the lateral right shin with central slough and surrounding erythema

On examination, there was a 4 cm x 5 cm ulcer on the lateral right shin with central slough and surrounding erythema (Figure 1).

**Figure 2:**
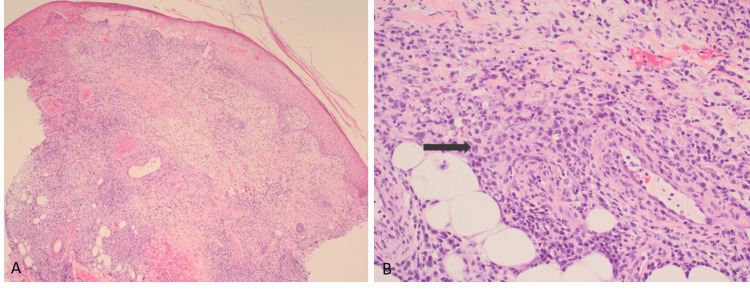
Lesional histopathology (A) A skin biopsy showing atypical lymphoid infiltrate in the dermis (Haemotoxylin & Eosin, original magnification x4) (B) The atypical infiltrate is composed of medium to large-sized lymphoid cells (denoted by arrow) and is seen extending into the subcutaneous fatty tissue (Haematoxylin & Eosin, original magnification x20)

**Figure 3:**
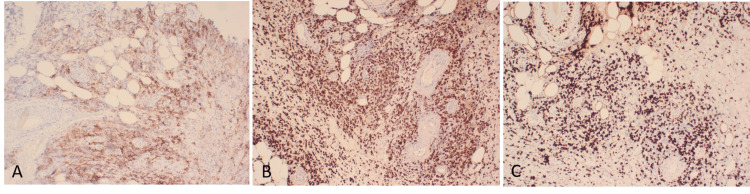
Immunohistochemistry staining The atypical lymphoid cells are positive for CD20 (A) and EBER-ISH (B) and show a high Ki67 proliferation index (C). All original magnification x20

The differential diagnoses included pyoderma gangrenosum or an atypical infection. An X-ray did not identify any evidence of osteomyelitis. A skin biopsy (Figure 2) showed an atypical lymphoid infiltrate in the dermis, extending to the subcutaneous fat, composed of medium to large-sized lymphoid cells with hyperchromatic nuclei and small amounts of cytoplasm, associated with karyorrhectic necrosis. Some of the small vessels in the dermis showed fibrinoid necrosis of the wall. Immunohistochemistry staining was performed (Figures 3A-3C). Atypical lymphoid cells were positive for PAX5 (expressed in B-cell neoplasms), B-cell marker CD20 (Figure 3A) and MUM1 (expressed in B-cells and activated T-lymphoid cells), but negative for CD3, BCL6, CD10, cyclin D1, C-MYC, CD4 and CD8. In-situ hybridisation for EBV was positive (Figure 3B). The Ki-67 cell proliferation index was high at 70% (Figure 3C). Tissue cultures for fungi and mycobacteria were negative. Serum EBV viral load was undetectable at < 500 copies per ml.

**Figure 4:**
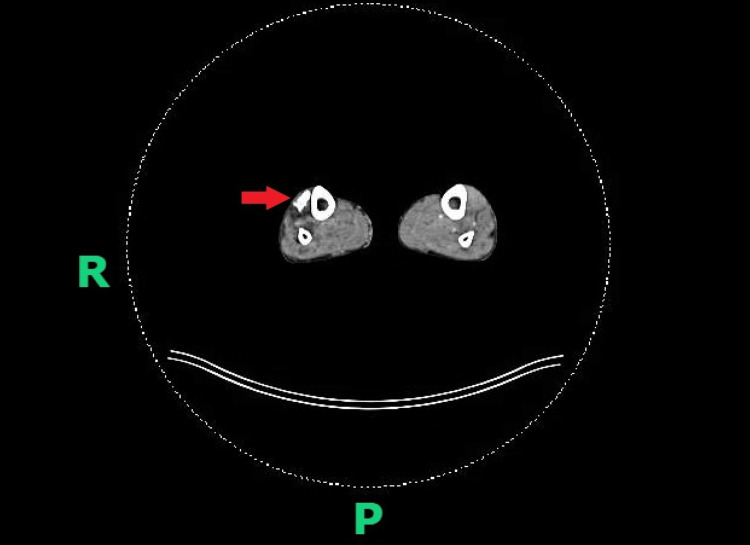
Whole-body fluorodeoxyglucose positron emission tomography scan Red arrow demonstrating increased tracer uptake on the lateral aspect of the right lower leg, corresponding to the site of the cutaneous ulcer. R (right), P (posterior)

Based on the history, skin biopsy and immunohistochemistry findings, our patient was diagnosed with monomorphic B-cell post-transplant lymphoproliferative disorder (PTLD) consistent with diffuse large B-cell lymphoma, non-germinal centre subtype. A whole-body fluorodeoxyglucose (FDG) positron emission tomography (PET) scan showed increased tracer uptake corresponding to the site of the cutaneous ulcer (Figure 4). In addition, three other sites showed increased tracer uptake (anterior cortex of the right lower tibia, an area adjacent to the right superficial femoral artery and the right inguinal node) and this was of uncertain significance, although the presence of PTLD remained a possibility.

Initially, azathioprine was stopped. The patient was commenced on intravenous anti-CD20 antibody, rituximab, which was administered weekly for eight weeks and was tolerated well. Clinical resolution of the ulcer was subsequently observed. A post-treatment FDG-PET scan demonstrated a negative signal in all the areas enhanced in the baseline scan, demonstrating a complete response to treatment [4]. To our knowledge, there has been no disease reoccurrence to date.

## Discussion

After squamous cell carcinoma, PTLD is the main cause of cancer-related death among solid organ transplant recipients [5]. According to the World Health Organisation classification [6], PTLD is divided into several categories: early lesions, monomorphic PTLD (B-cell, T-cell or NK cell), polymorphic PTLD and classic Hodgkin’s lymphoma type. Our patient had monomorphic PTLD, which is a more aggressive subtype [7] and typically responds poorly to a reduction in immunosuppression. Our case is unusual because although PTLD is heterogeneous in presentation and extra-nodal sites are commonly affected, cutaneous involvement tends to be much rarer with only 5% of cases of PTLD reported to affect the skin [8], and only a handful of such cases reported to date. Cutaneous manifestations of PTLD are highly variable, ranging from papules to nodules with ulceration, as well as maculopapular eruptions [9,10]. Affected sites can include the face, trunk and lower limbs. Our patient initially presented with a painless ulcer on the leg, which appeared relatively innocuous, thus highlighting the need to maintain a low threshold to investigate any new cutaneous lesion in a transplant recipient.

PTLD can be classified as early-onset, which occurs less than a year following transplantation, or late-onset, occurring at least one year later. Late-onset disease is not uncommon with PTLD, including cutaneous involvement and can occur many years after a transplant, with a reported occurrence after 15 years [11]. There is an increased risk of PTLD for those transplants associated with significant immunosuppression such as lung, heart and intestine when compared to renal transplantation [12]. Other risk factors include EBV infection (mostly associated with early-onset disease), advancing age and immunosuppressive agents, in particular calcineurin inhibitors ciclosporin and tacrolimus [13,14]. Mortality rates have been reported to be around 60% and higher in older patients, those with monomorphic tumours and disease with later onset [15].

Treatment of PTLD depends on the subtype of the disease as well as its severity. Reduction in immunosuppression tends to be a universal first step in managing PTLD though this must be balanced against the risk of transplant rejection. For our patient, azathioprine was initially stopped, but additional treatment was required due to a more aggressive subtype. Treatment options include rituximab monotherapy or combined with CHOP (cyclophosphamide, hydroxydaunorubicin, vincristine and prednisolone) chemotherapy and radiotherapy [16,17]. When disease is confined to the skin, outcomes tend to be more favourable [13] than when there is systemic involvement.

## Conclusions

In summary, we report a rare case of an aggressive form of PTLD in a patient with a cardiac transplant, presenting with a painless leg ulcer. PTLD seldom affects the skin and is highly variable in presentation, yet can be aggressive and potentially fatal. It can also present many years post-transplant. Treatment with rituximab resulted in disease resolution. Therefore, it is important for dermatologists to consider PTLD in their differential diagnoses so that prompt diagnosis and treatment can ensue.
